# Supplementary material for: Empirical methods for controlling false positives and estimating confidence in ChIP-Seq peaks
Source: BMC Bioinformatics. 2008 Dec 5;9:523. doi: 10.1186/1471-2105-9-523 (PMC2628906; doi:10.1186/1471-2105-9-523)
Supplement: Additional File 1 — A variety of html documents from the USeq web site detailing the available applications, their best usage, output file type descriptions, command line menus, etc. [file 1471-2105-9-523-S1.zip › USeqUserGuides/JavaDoc.zip!/JavaDoc/edu/utah/seq/methylation454/CpG.html]

CpG


|  |  |  |
| --- | --- | --- |
| **Overview** \| **Package** \| **Class** \| **Use** \| **Tree** \| **Deprecated** \| **Index** \| **Help** | |  |
| **PREV CLASS**   **NEXT CLASS** | **FRAMES**    **NO FRAMES**     **All Classes** |  |
| SUMMARY: NESTED \| FIELD \| CONSTR \| METHOD | DETAIL: FIELD \| CONSTR \| METHOD |  |


---


## edu.utah.seq.methylation454 Class CpG

```
java.lang.Object
  edu.utah.seq.methylation454.CpG
```

---

``` public class CpG extends java.lang.Object ```

---

**Constructor Summary**

`CpG(java.lang.String id)`


| **Method Summary** | |
| --- | --- |
| `double` | `fractionC()` |
| `java.lang.String` | `getId()` |
| `int` | `getNumberCs()` |
| `int` | `getNumberReads()` |
| `BaseRead[]` | `getReads()` |
| `void` | `setReads(BaseRead[] reads)` |
| `java.lang.String` | `toString()` |

**Methods inherited from class java.lang.Object**

`equals, getClass, hashCode, notify, notifyAll, wait, wait, wait`

**Constructor Detail**

### CpG

```
public CpG(java.lang.String id)
```


**Method Detail**

### toString

```
public java.lang.String toString()
```

:   **Overrides:**: `toString` in class `java.lang.Object`

---


### getId

```
public java.lang.String getId()
```

---


### getReads

```
public BaseRead[] getReads()
```

---


### setReads

```
public void setReads(BaseRead[] reads)
```

---


### getNumberReads

```
public int getNumberReads()
```

---


### fractionC

```
public double fractionC()
```

---


### getNumberCs

```
public int getNumberCs()
```


---


|  |  |  |
| --- | --- | --- |
| **Overview** \| **Package** \| **Class** \| **Use** \| **Tree** \| **Deprecated** \| **Index** \| **Help** | |  |
| **PREV CLASS**   **NEXT CLASS** | **FRAMES**    **NO FRAMES**     **All Classes** |  |
| SUMMARY: NESTED \| FIELD \| CONSTR \| METHOD | DETAIL: FIELD \| CONSTR \| METHOD |  |


---
